# The downsizing of gigantic scales and large cells in the genus *Mallomonas* (Synurales, Chrysophyceae)

**DOI:** 10.1038/s41598-022-09006-1

**Published:** 2022-03-22

**Authors:** Peter A. Siver

**Affiliations:** Department of Botany, Connecticut College, New London, CT 06320 USA

**Keywords:** Palaeontology, Biodiversity, Freshwater ecology, Limnology

## Abstract

*Mallomonas* is the largest and most speciose genus within the Synurales, a monophyletic clade of siliceous scale-bearing organisms within the class Chrysophyceae. The genus consists of unicellular, motile, photosynthetic organisms found in freshwater localities worldwide. *Mallomonas* diverged from other synurophytes during the lower Cretaceous at approximately 130 Ma. Recent discoveries of fossil species were used to examine shifts in scale and cell size over geologic time. On average, scales of fossil species were 2.5 times larger than those produced by modern species. However, a smaller subset of extinct fossil taxa lacking modern analogs had scales over four times larger than modern species, and the largest recorded specimens were six times larger. Data from modern species were further used to develop a model relating scale size to cell size, and applied to the fossil specimens. Based on the model, the mean size of fossil cells was almost twice as long and 50% wider compared to modern species, and cells of taxa lacking modern analogs close to three times as large. These large cells, covered with robust siliceous scales, were likely slow swimmers requiring significant energy to maintain their position in the water column, and possibly prone to increased predation.

## Introduction

*Mallomonas* Perty is a genus of photosynthetic, unicellular, motile, heterokont algae belonging to the order Synurales, a monophyletic clade of siliceous scale-bearing organisms nested within the class Chrysophyceae^1–4^. The genus is the largest and most speciose within the Synurales, distributed worldwide, with most of the species inhabiting the planktic community of freshwater ponds and lakes^1,3,5^. Although limited in cold polar regions^1,5^, high diversities of *Mallomonas* species have been reported over a wide range of subarctic, temperate, subtropical and tropical localities^3,7–10^.

The richest floras of *Mallomonas* species tend to be found in waterbodies that are slightly acidic, low in specific conductance, and with moderate concentrations of nutrient and dissolved humic substances [^3^, and references therein]. Many species have restricted distributions along physical and chemical gradients, making them excellent bioindicators^3^. The differential distributions of species along environmental gradients, coupled with their species-specific siliceous components becoming preserved in sediments, has resulted in the genus being useful in reconstructing past lakewater conditions^6,11,12^, and for examination of evolutionary history^13^.

As is true of all Synurales, *Mallomonas* species are characterized by an outer layer comprised of siliceous scales that are precisely arranged to form a highly organized cell covering^1,3,14^. Many species form differently shaped scales that fit in precise locations on the cell surface, for example ones that align the flagellar pore, cover the main body, or surround the posterior end. Regardless of where the scales fit within the cell covering, the basic design and ornamentation is similar and diagnostic at the species level. In addition to scales, almost all species of *Mallomonas* possess a second type of siliceous structure, the bristle. Bristles are thin, elongate structures with one end, called the foot, modified to fit under the apical end of a scale such that the long shafts radiate outwards from the cell.

**Figure 1:**
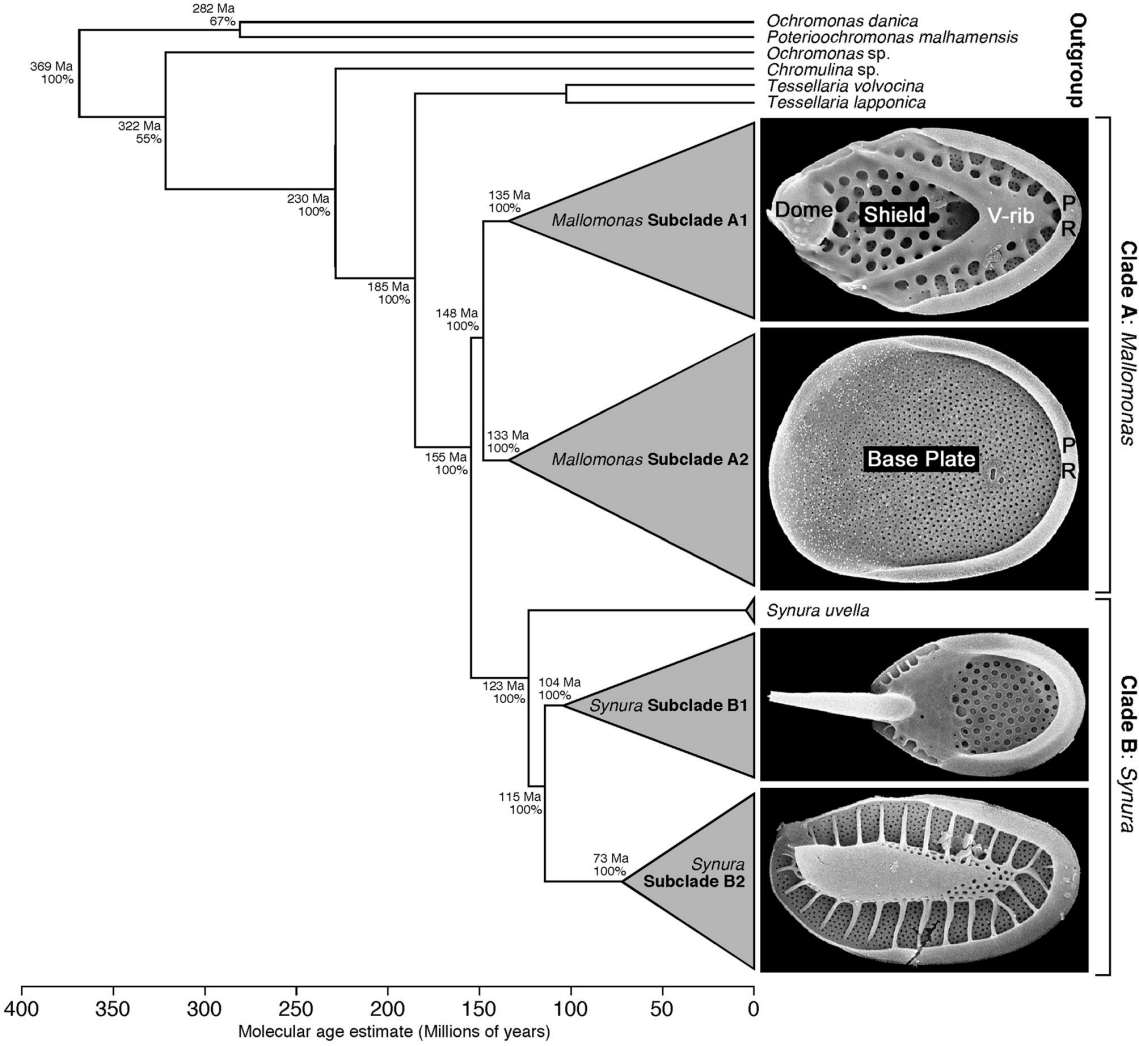
Time-calibrated phylogenetic tree for the Synurales based on a five-gene data set after Siver et al.^19^. The mean divergence time (top number in millions of years) and percent posterior probability (bottom number) are given at each node. Clades representing *Mallomonas* (clade A) and *Synura* (clade B), along with major subclades for each genus are illustrated with representative scale types. For *Mallomonas*, subclades A1 and A2 include taxa that have scales with and without a V-rib, respectively. Primary parts of each *Mallomonas* scale type are denoted, including the dome, shield, V-rib, base plate and posterior rim (PR). Reprinted with permission from the Botanical Society of America.

At a minimum, all scales possess a base plate that is perforated, at least in part, with small pores, and a posterior upturned rim, known as the posterior rim, that bends up and over the base plate along the proximal margin (Fig. 1); this is the basic scale design for species in section Planae^1,15,16^. Most species have scales with additional secondary layers of silica deposited onto the base plate that form distinctive designs. Other species produce more complex scales that possess structures such as a V-rib, dome, spines or wings. As the name implies, the V-rib is a V-shaped ridge of silica positioned on the base plate, and forming the boundary between the shield and the posterior flange (Fig. 1). The V-rib is viewed as a structure that aids in orientating and spacing the scales, and those species with such scales typically have a close-fitting and precise cell covering^3,14^. The dome is a raised cavity on the distal end of the scale into which the bristle foot, is tucked. The bristle shaft emerges from an inverted U-shaped opening along the distal margin of the dome. Scales that possess a dome and V-rib are termed tripartite scales because they have three regions, the dome, shield and posterior flange^17^.

Until the recent discovery of numerous synurophyte remains in the early Eocene Giraffe Pipe fossil locality, there was no record of this group of organisms older than Holocene^18,19^. Findings from this fossil locality have yielded preliminary insights into how scales, bristles and cells have evolved over time, and have aided in linking evolutionary events to geologic time^20–22^. Using relaxed molecular clock methods calibrated with Giraffe microfossils, Siver et al.^19^ estimated the origin of the synurophytes to be upper Jurassic, approximately 156 Ma, with the primary genera *Mallomonas* and *Synura* diverging from each other during the lower Cretaceous at 130 Ma (Fig. 1). A Cretaceous timeframe for divergence of *Mallomonas* and *Synura* was supported by Skaloud et al.^23^. Jo et al.^24^ and Siver et al.^19^ further showed that *Mallomonas* diverged into two major clades during the Cretaceous, one clade consisting of species with scales lacking a V-rib (Fig. 1; subclade A2), and the other with V-rib-bearing scales (Fig. 1; subclade A1).

Siver et al.^19^ offered preliminary findings regarding shifts in the sizes of scales since the Eocene, including identification of some fossil species bearing significantly larger scales compared to those formed by modern species. The objectives of this study are to (1) further use the fossil record to examine shifts in the size of *Mallomonas* scales and; (2) establish a model relating scale size to cell size, and use it to estimate the sizes of fossil *Mallomonas* cells.

## Materials and methods

The morphometric database of scale and cell sizes used in this study was developed from one originally used by Siver et al.^19^. The initial database consisted of data for 100 modern species of *Mallomonas* that were taken from Siver^15^ and Kristiansen^16^. Scale and cell morphometric data for an additional 24 modern species described after the Kristiansen^16^ publication were taken from original descriptions and literature records, and scale data for 21fossil species (Table 1), were added to the database for this study, resulting in a total of 145 taxa (Supplementary File 1). Seventeen of the 21 fossil species have now been officially described (Table 1). Three of the remaining four fossil species, *Mallomonas* GP2, *M*. GP4 and *M*. GP13, were included in Siver et al.^19^, and *Mallomonas* W1is illustrated for the first time in this study. *Mallomonas* W1 was uncovered from the 83 Ma old Cretaceous Wombat locality^25^, and the remaining fossil taxa from the 48 Ma Eocene Giraffe pipe locality^19^.

**Table 1:** List of 21 fossil *Mallomonas* species used in this study. All species except *Mallomonas* W1, which was found in the Cretaceous Wombat locality, were uncovered from the Giraffe Pipe fossil locality. Each species is scored as a) being similar to a modern taxon; b) closely related to a modern taxon or; c) lacking a modern analog. Section assignments within the genus are according to Kristiansen^16^ or as modified by Siver et al.^19^, subclade assignments after Siver et al.^19^ and as shown in Fig. 1, and those species reported as stem lineages are noted. See text for details. Table modified and updated from Table 1 in Siver et al.^19^.

Species	Modern Analog			Section/Subclade/Stem	Publication
	Similar	Closely related	Lacking		
*Mallomonas*					
M. *bakeri* Siver		X		^1^Planae/A2	Siver^37^
*M. elephantus* Siver & Wolfe			X	Planae /A2/Stem	Siver and Wolfe^38^
*M*. *media* Siver & Lott			X	Planae/A2/Stem	Siver and Lott^39^
*M*. *pleuriforamen* Siver et al		X		Planae/A2	Jo et al.^24^
*M*. *porifera* Siver & Wolfe			X	Planae/A2/Stem	Siver and Wolfe^40^
*M*. *pseudocaudata* Siver & Wolfe		X		PlanaeA2	Siver and Wolfe^40^
*M. pseudohamata* Siver & Wolfe		X		^1^PlanaeA2	Siver and Wolfe^40^
*M*. *schumachii* Siver			X	Planae /A2/Stem	Siver^41^
*M. skogstadii* Siver			X	^1^Planae/A2/Stem	Siver^37^
*Mallomonas* GP13			X	Planae/A2/Stem	^3^Siver et al.^19^
*Mallomonas* W1			X	Planae /A2/Stem	^2^This paper
*Mallomonas* GP4		X		Punctiferae/A1	^3^Siver et al.^19^
*M*. *lychenensis* Conrad	X			Quadratae/A1	Kristiansen^16^
*Mallomonas* GP2	X			Torquatae/A1	^3^Siver et al.^19^
*M*. *preisigii* Siver			X	Papillosae (?)/A1	Siver and Lott^39^
*M*. *ampla* Siver & Lott	X			Multisetigera/A1	Siver and Lott^39^
*M. aperature* Siver		X		Leboimianae/A1	Siver^22^
*M*. *giraffensis* Siver & Wolfe		X		Pseudocoronatae/A1	Siver and Wolfe^40^
*M*. *dispar* Siver, Lott & Wolfe		X		Mallomonas/A1	Siver et al.^20^
*M*. *lancea* Siver, Lott & Wolfe		X		Mallomonas/A1	Siver et al.^20^
*M*. *convallis* Siver & Wolfe			X	?/A1	Siver and Wolfe^40^

^1^According to Jo et al. (2011) the section Heterospinae should be combined with section Planae.

^2^Taxon first illustrated in this paper.

^3^Taxon first illustrated in Siver et al.^19^, but not yet formally described.

Except for data given in Siver^15^, literature records for most modern *Mallomonas* species include only ranges and not mean values. In lieu of the lack of mean estimates for most modern species, range midpoints for scale and cell data were used to develop models relating scale size to cell size. Range midpoint values were also used to estimate cell size for fossil species. Between 20–30, and a minimum of ten, scales were measured for each fossil species. As reported by Siver et al.^19^, the fact that there are highly significant relationships between mean and range midpoint measurements for the suite of species where both values are available, supports the use of range midpoints as a surrogate for mean values. As a result, the mean values of cell size reported in the study are equivalent to range midpoints.

Scale surface area was estimated using the formula for an ellipse: SA = (a * b * π), where a = radius of the major axis, b = radius of the minor axis, and π = 3.14. As the diameter of the minor axis approaches that of the larger axis, the formula converges on the surface area of a circle, and 78.5% of the area of a true square. Thus, it works well for estimating surface area of scales that are ellipse-shaped, and those reported as circular-shaped. In addition, this formula provides a reasonable estimate of surface area for scales reported as “square-shaped,” since these scale types actually form rounded margins and not right-angled edges.

Regression analyses were used to relate scale length, width and surface area measurements, and for exploring the relationships between scale size and both cell length and width. Relationships between any two scale size measurements were found to be highly correlated, and all three estimates of scale size were significantly related to cell size. Because cell length was most highly correlated with scale surface area, and cell width with scale length, these models were used to estimate cell length and width for all fossil species. Because of the high correlation between any combination of scale size parameters (e.g. scale length and width), adding multiple independent variables did not significantly improve either model for estimating cell size. Linear regression analyses were done using SigmaPlot v. 12.5.

## Results

### Scale size

**Figure 2:**
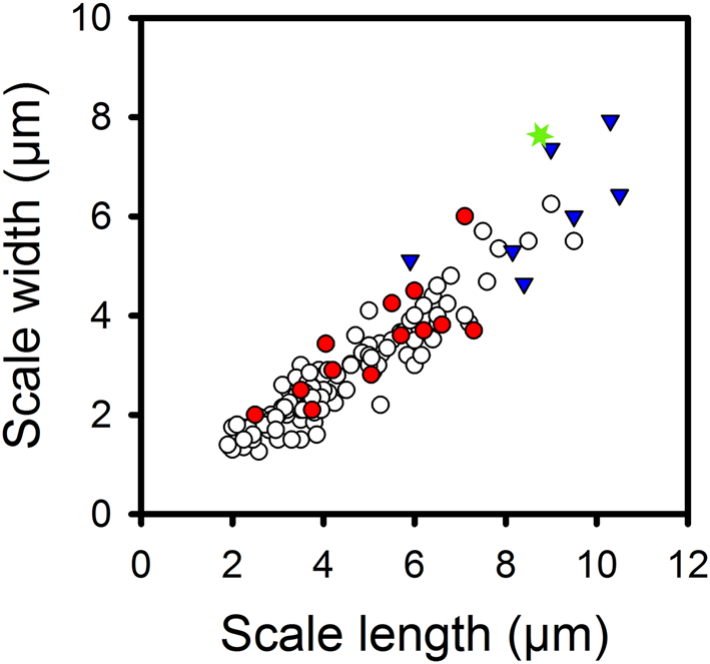
Relationship between scale length and scale width for 145 *Mallomonas* species, including 124 modern and 21 fossil species. Key: modern species = open circles; fossil species from the Giraffe fossil locality with modern analogs = red circles; fossil species from the Giraffe fossil locality lacking modern analogs = blue triangles; and the oldest known fossil *Mallomonas* species from the Wombat fossil locality = green star. The r^2^ = 0.88; *p* < 0.0001.

**Figure 3:**
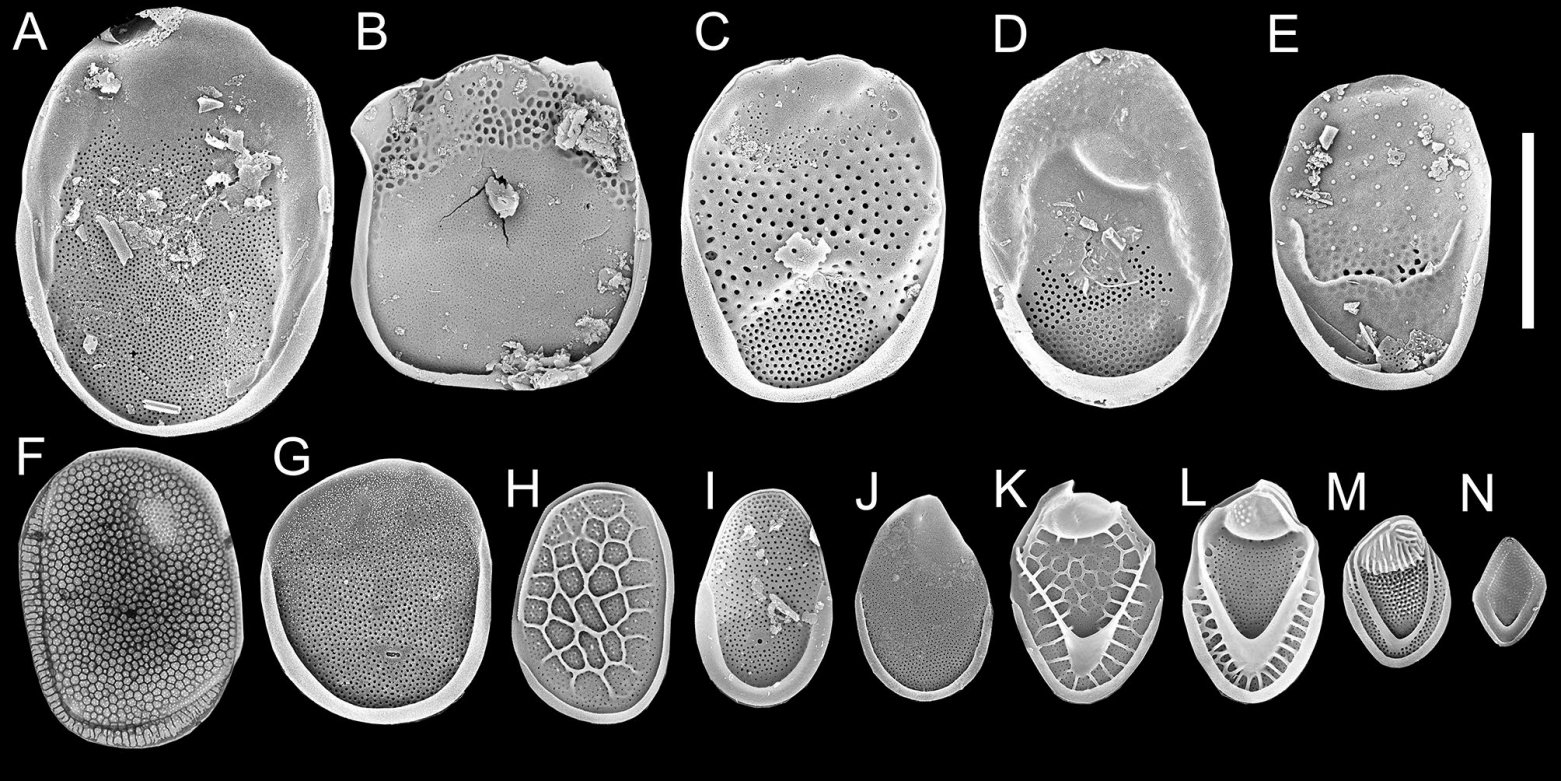
Representative body scales of five fossil (top row,** A**-**E**) and nine modern (bottom row,** F**-**N**) *Mallomonas* species illustrated at the same scale (bar = 5 µm). (**A**) *M*. GP13; (**B**) *M*. W1; (**C**) *M. schumachii*; (**D**) *M*. *media*; (**E**) *M. elephantus*; (**F**) *M*. *bronchartiana*; (**G**) *M*. *caudata*; H) *M*. *teilingii*; (**I**) *M. matvienkoae*; (**J**) *M*. *hamata*; (**K**) *M*. *crassisquama*; (**L**) *M*. *muskokana*; (**M**) *M*. *wujekii* and; (**N**) *M*. *torquata* f. *simplex*. The fossil taxa are believed to represent stem taxa in *Mallomonas* subclade A2 (see Fig. 1) represented by section Planae. Specimens illustrated in (**F**)-(**J**) represent scales of modern species belonging to section Planae (as modified by Jo et al.^24^), and those pictured in (**K**)-(**N**) modern species in subclade A1 that possess a V-rib.

The mean length and width of body scales for modern species (n = 124) ranged from 1.9–9.5 µm and 1.3–6.2 µm, and for fossil species (n = 21) from 2.5–10.5 µm and 2–7.9 µm, respectively (Table 2). The overall mean scale size of modern species was 4.2 × 2.7 µm, yielding a mean surface area of 10.1 µm^2^. In contrast, the mean scale size of the fossil taxa was 6.6 × 4.6 µm, yielding a significantly larger scale surface area of 26.2 µm^2^ (Table 2). Fossil taxa lacking modern representatives (n = 9) had a mean scale size of 8.8 × 6.4 µm, resulting in an average scale surface area four times larger than modern species, and almost twice as large as fossil taxa with modern representatives (Table 2; Figs. 2, 3). Scale width is highly and significantly correlated with scale length, for modern taxa (r^2^ = 0.88, *p* < 0.001), fossil taxa (r^2^ = 0.81; *p* < 0.001) and both groups combined (r^2^ = 0.88; *p* < 0.001; Table 3).

**Table 2:** Mean, minimum and maximum estimates of scale length, width and surface area, and cell length and width. Estimates are given for modern species, all fossil species, and fossil species that lack modern congeners.

Category	Variable	Mean ± SD	Minimum	Maximum
Modern species	Scale length (µm)	4.2 ± 1.6	1.9	9.5
	Scale width (µm)	2.7 ± 1.0	1.3	6.2
	Scale surface area (µm^2^)	10.1 ± 8.1	2.0	44.2
	Cell length (µm)	21.4 ± 10.8	7	60
	Cell width (µm)	9.8 ± 4.2	2.5	21
All fossil species	Scale length (µm)	6.6 ± 2.3	2.5	10.5
	Scale width (µm)	4.6 ± 1.8	2.0	7.9
	Scale surface area (µm^2^)	26.2 ± 17.7	3.9	64.1
	Cell length (µm)	39.2 ± 19.8	14.3	81.7
	Cell width (µm)	14.9 ± 4.8	6.4	23.2
*Fossil species	Scale length (µm)	8.8 ± 1.4	5.9	10.5
	Scale width (µm)	6.4 ± 1.2	4.6	7.9
	Scale surface area (µm^2^)	44.2 ± 13.6	23.7	64.1
	Cell length (µm)	59.4 ± 15.2	36.4	81.7
	Cell width (µm)	19.6 ± 3.0	13.5	23.2

*Fossil species lacking modern congeners.

**Table 3:** Results of linear regression analyses relating scale and cell size for species of the synurophyte genus, *Mallomonas*. All length and width units are in µm, and surface area measurements in µm^2^.

Independent variable (x)	Dependent variable (y)	Linear model	r^2^	*p* value
^1^Scale length	Scale width	y = 0.141 + (0.605 * x)	0.88	*p* < 0.0001
^2^Scale length	Scale width	y = 0.019 + (0.643 * x)	0.88	*p* < 0.0001
Scale surface area	Cell length	*y = 9.88 + (1.12 * x)	0.74	*p* < 0.001
Scale length	Cell length	y = − 3.1 + (5.67 * x)	0.71	*p* < 0.001
Scale surface area	Cell width	y = 6.1 + (0.37 * x)	0.50	*p* < 0.001
Scale length	Cell width	*y = 1.1 + (2.1 * x)	0.59	*p* < 0.001

^1^Model for modern species; ^2^ model for modern and fossil species combined; *models used to estimate cell sizes of fossil species.

Of the nine extinct fossil species lacking a clear link to modern taxa, seven would be classified within the section Planae (Fig. 1; *Mallomonas* subclade A2). Five of the seven have body scales that are among the largest known for the genus (Figs. 2, 3A-E), and several tend to be slightly square to rectangular-shaped (e.g. Fig. 3B-C). For example, scales of *Mallomonas* GP13 (Fig. 3A) are the largest recorded, with a surface area over six times larger than the average for modern *Mallomonas* species, and 2–2.5 × greater than the largest known modern representatives (e.gs. Fig. 3F-G). Body scales of *Mallomonas* Wsp1, the oldest recorded species in the genus, are distinctively square-shaped and five times larger than the average for modern species (Fig. 3B). Scales of *M*. *schumachii* (Fig. 3C) and *M*. *media* (Fig. 3D) are also massive and robust, with surface areas 4–5 times larger than the average for modern species. Although body scales of the extinct *Mallomonas elephantus* (Fig. 3E) had a similar surface area to those of *M*. *bronchartiana* (Fig. 3F), the modern species with the largest scales belonging to section Planae, many scales of the former taxon also possessed a large wing-like structure protruding to one side on the scale. Scales of the two remaining extinct fossil species within section Planae, *Mallomonas skogstadii* and *M*. *porifera*, were also large with mean values of 8.4 × 4.7 µm and 5.9 × 5.1 µm, respectively. As is the case for *M*. *elephantus*, scales of *M*. *skogstadii* have a large protruding anterior wing. Scales of *M*. *porifera* have a similar surface area to those of the modern species *M*. *caudata* (Fig. 3G), but with a distinctly circular shape. In addition to size and shape comparisons of scales of modern species in section Planae (Fig. 3F-J), representatives of common species representing *Mallomonas* subclade A1 (Fig. 1) with tripartite scales (Fig. 3K-M) and a species from section Torquatae (Fig. 3N) further emphasize the large size of the extinct fossil species.

### Cell size

**Figure 4:**
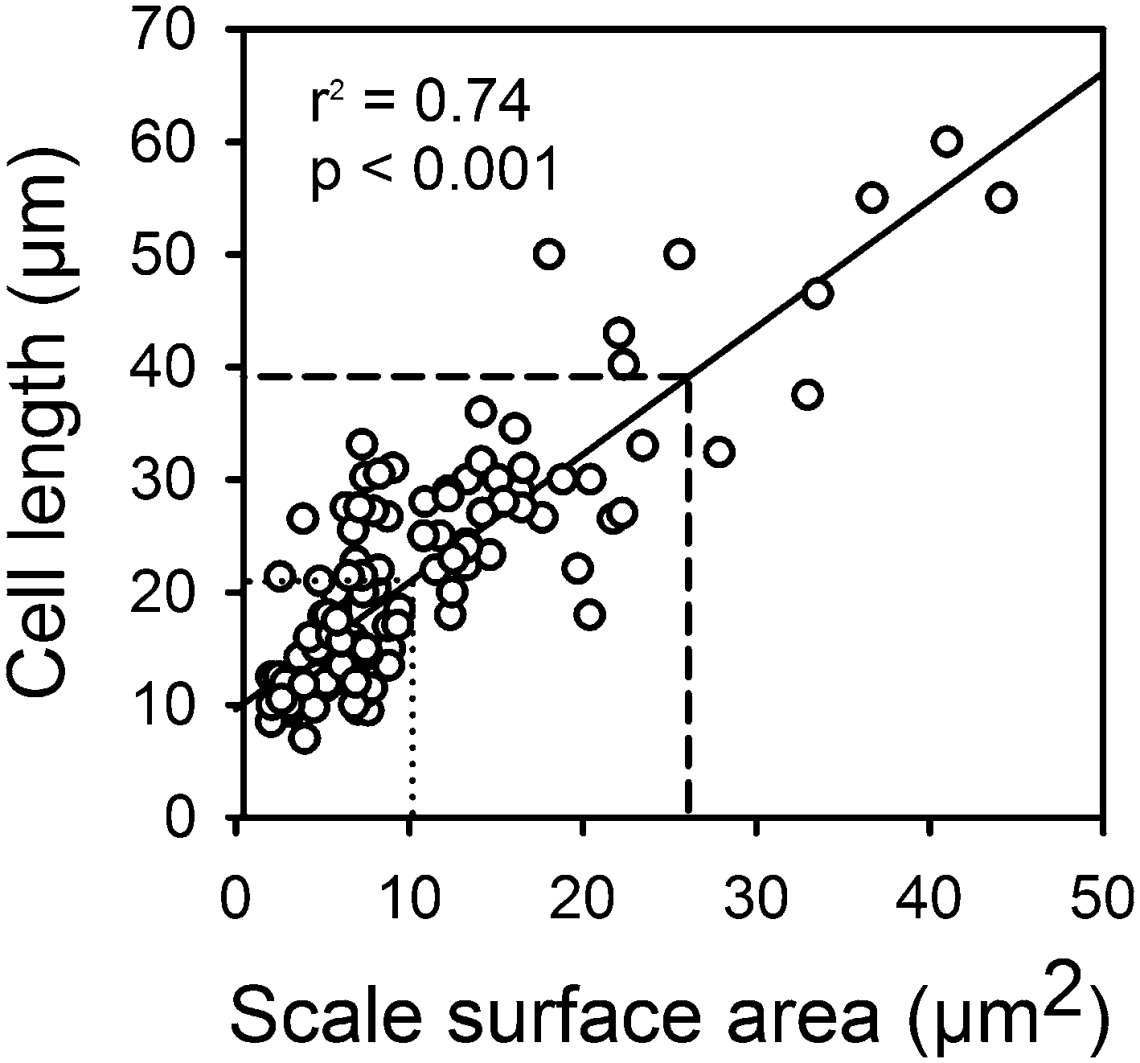
The relationship between scale surface area and cell length based on data from 124 modern *Mallomonas* species (r^2^ = 0.74; *p* < 0.001). The dotted lines represent the overall mean for modern species, and the dashed lines the estimated mean for fossil species. Note that the model estimates the smallest *Mallomonas* cell to be 9.9 µm, close to the mean of 9.3 µm for the five smallest modern species.

**Figure 5:**
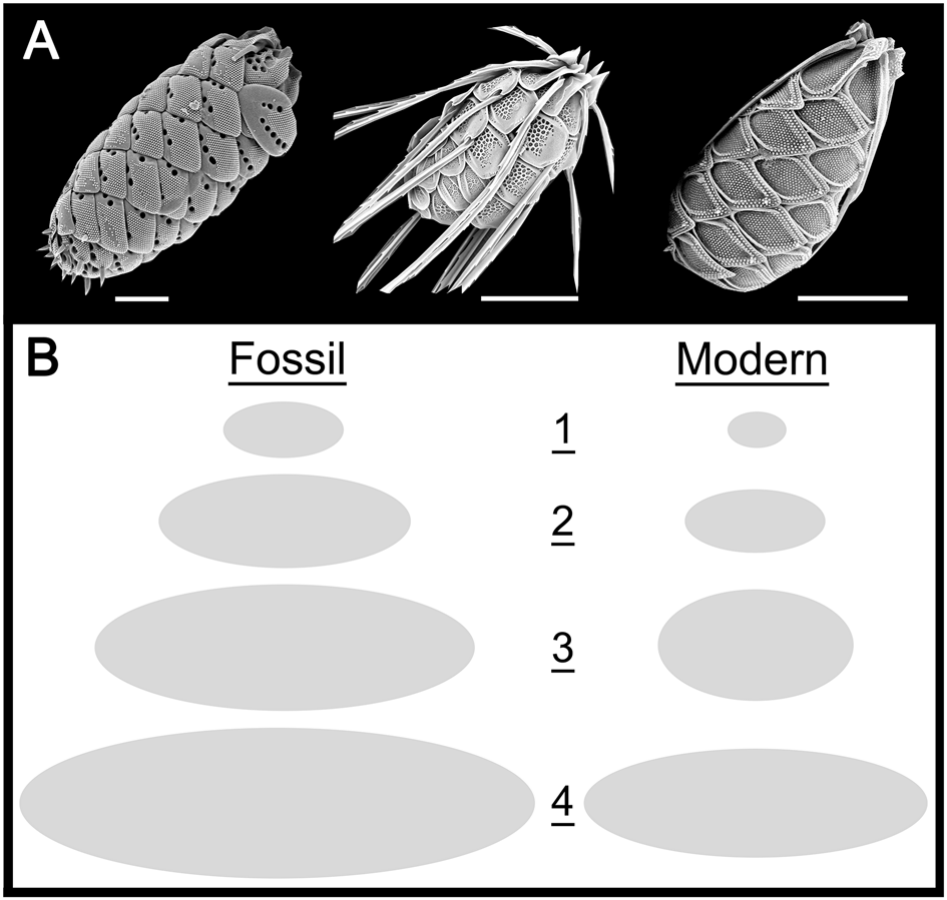
(**A**) Whole cells of three modern *Mallomonas* species, *M*. *lychenensis*, *M*. *punctifera* and *M*. *mangofera* from left to right. Scale bar = 5 µm. (**B**) Relative sizes of fossil versus modern cells for (1) the five smallest species in each group; (2) means for all fossil and modern species in the dataset; (3) means for fossil species lacking modern congeners, and five of the larger modern species within section Planae and; (4) the largest species in each group. See text for details.

The mean lengths and widths of cells of modern species (n = 122) ranged from 7–60 µm, and 2.5–21 µm, with overall mean and median values of 21.4 × 10.8 µm, and 18.0 × 9.5 µm, respectively (Table 2; Fig. 4). Although cells of some species tend to be more spherical-shaped, the vast majority are ellipsoidal-shaped (Fig. 5A). Only four species, *M*. *caudata*, *M*. *bronchartiana*, *M*. *insignis* and *M*. *leboimei*, have a mean cell length ≥ 50 µm, and three a mean width ≥ 20 µm (Supplementary File 1).

The relationship between scale size and cell size of modern species was investigated for the purpose of potentially reconstructing the cell size of fossil taxa using scale size data. Both cell length and cell width were found to be significantly correlated with scale size (Table 3). Cell length was most highly correlated with scale surface area (r^2^ = 0.74; *p* < 0.001; Fig. 4), and to a slightly lesser extent with scale length (r^2^ = 0.71; *p* < 0.001) (Table 3). Cell width was slightly more correlated with scale length (r^2^ = 0.59; *p* < 0.001) than scale surface area (r^2^ = 0.50; *p* < 0.001) (Table 3).

The linear models relating scale surface area to cell length, and scale length to cell width, were applied to the fossil taxa in order to estimate the cell sizes of these ancient organisms (Table 2). The estimated cell length and width of the fossil species ranged from 14.3–81.7 µm and 4.8–23.2, respectively, with a mean cell size of 39.2 × 14.9 µm (Fig. 4). The mean length of the fossil cells was almost double that of the modern species, and 50% wider (Fig. 5B2). The mean size of cells for fossil taxa that lack modern congeners was significantly larger with a mean of 59.4 × 19.6 µm, and with cells of *M*. GP13 reaching an estimated 81.7 × 22.7 µm (Fig. 5B4). This is in contrast to a mean cell size of 31.0 × 18.5 µm for the five most common modern species belonging to section Planae, and a cell size of 55.0 × 19.0 µm for the largest modern species in section Planae, *M*. *bronchartiana* (Fig. 5B4). Lastly, the mean size of the five smallest fossil species was 18.0 × 8.7 µm, compared to a mean of 9.3 × 5.0 µm for the five smallest modern taxa.

## Discussion

Siver et al.^19^ identified three categories of fossil *Mallomonas* species uncovered in the extensive Giraffe Pipe locality. One group of species had scales with morphological characteristics similar to, and difficult to separate from, modern congeners. Based on a morphological species concept, these could be viewed as representing the same species. A second group had morphologically different scales, but ones that could be linked to one or more modern species. The third group possessed scales that could not be directly linked to any modern species. The majority of the species contained in the latter group lacked a V-rib and well developed dome, and were considered as stem organisms within the broad section Planae. Siver et al.^19^ further reported that the mean size of scales in the group containing the extinct stem taxa was larger than those fossil taxa grouped with modern congeners.

The current study adds additional modern and fossil species to the database used by Siver et al.^19^, including the oldest known taxon from the Cretaceous Wombat locality, and provides the first attempt to reconstruct cell size for fossil *Mallomonas* species. Based on the expanded database, several trends with respect to the evolution of scale and cell size of *Mallomonas* taxa can be made. First, there is a strong relationship between scale width and scale length that was similar for both fossil and modern forms. Second, as a group, fossil taxa had scales that are significantly larger than those produced by modern species, especially with respect to surface area. The five species with the largest scales belong to extinct fossil species, four of which belong to the group of stem taxa within section Planae. These scales are massive compared with modern forms, and support the concept of scale gigantism for early members of the *Mallomonas* clade containing species with scales that lack a V-rib and dome (Fig. 1; subclade A2). Third, assuming the model relating scale and cell size can be applied to the geologic record, fossil species produced significantly larger cells than modern forms.

Because the models relating scale length to scale width were similar for modern and fossil species, the assumption is that the models developed relating scale size to cell size are appropriate for fossil taxa. In addition, the precise overlapping pattern of scales comprising the cell covering on modern species has recently been documented for Eocene fossil species^22^, indicating that this architectural design was well evolved by at least the early Eocene. Thus, making the assumption that other fossil taxa had similarly constructed cell coverings is reasonable, and further supports the application of the models relating scale and cell size to these fossil forms.

Based on the model estimates, the mean cell size of the fossil species is approximately twice as large as the average cell produced by modern organisms. This doubling of cell size was also observed for the smallest species. The mean size of the five smallest modern species (*M*. *canina*, *M*. *mangofera*, *M*. *dickii*, *M*. *madagascariensis*, and *M*. *gutata*) was 9.3 × 5 µm, compared to the mean cell size estimated for the five smallest fossil taxa (*M*. *pseudohamata*, *M*. *preisigii*, *M*. *dispar*, *M*. *bakeri* and *M*. GP4) of 18 × 8.7 µm. The cell size discrepancy is even greater for fossil species that lack modern congeners, and especially for the extinct stem species within section Planae that possessed an average cell size of 69.2 × 20.8 µm, with a maximum cell size of 81.7 × 22.7 µm for *M*. GP13. The scales produced by these large fossil cells were not only massive in size, but also robust and heavily silicified. It is likely that these large cells covered with large, heavy and cumbersome scales would have been slow swimmers that expended significantly more energy to maintain their position in the water column than modern species. Perhaps these cells were also more prone to predation by larger zooplankton, and a combination of decreased motility and greater predation provided the evolutionary pressure for smaller and faster cells with less dense siliceous components, and ultimately caused the demise of the large-celled fossil species. In contrast, it is also possible that the stimulus initially resulting in the evolution of the larger species was the fact that they were too big to be preyed upon by smaller invertebrates.

Several points regarding the models used to estimate cell size are warranted. First, it is important to note that because the scale sizes used to estimate cell sizes for the larger fossil taxa are at the end of the range used to produce the model, caution needs to be exercised. The assumption is being made that the linear relationship of the model holds for the larger scales, and that the linear relationship does not begin to flatten and reach a maximum cell size. However, there is no indication that the relationship is reaching an asymptote, nor reason to assume that the model would not hold for organisms that produce larger siliceous components. Second, the scale and cell size data used to produce the models consisted of the midpoint values of the ranges given in the literature. Thus, the cell sizes inferred from the models represent a midpoint estimate of the range for each species, and not an upper size limit. Third, there is more data available in the literature documenting scale size than there is for cell size for most modern *Mallomonas* species. Additional data on cell size, especially inclusion of mean values, may help to further fine-tune the models. Lastly, the formula of an ellipse was used to estimate scale surface area for the few species with “square-shaped” scales. Although this may slightly underestimate the surface area, using a formula for a square or rectangle would have resulted in an overestimation. Because the few species with square-shaped scales were primarily the extinct fossil taxa lacking modern congeners, their cell size may actually have been slightly larger than estimated in this study.

Interestingly, fossil scales that have morphologically similar (identical) modern counterparts were not significantly different in size from each other, implying that their corresponding cells were also of similar size. These taxa have significantly smaller scales compared to those species with gigantic scales, and closer to the mean of modern species. Perhaps, this is why the lineages of these morphologically-identical species have survived for tens of millions of years. Despite maintaining virtually identical scale types, the degree of genetic difference from a physiological or reproductive perspective between taxa with virtually identical siliceous components remains unknown^19,23^.

The extinct scale types are not only significantly larger than those of species with modern congeners, but some have a tendency of being more rectangular to square-shaped. In contrast, fossil scale types that can be linked to modern species, along with their contemporary counterparts, tend to have elliptical-shaped scales. This is especially true of body scales^15,16,19^. Although a few smaller species of *Mallomonas* form spherical cells, the vast majority of species produce ellipsoidal-shaped cells, and this is especially true of species forming larger cells^15,16^. Smaller elliptical-shaped scales would be more efficient in covering a curving ellipsoidal cell surface than larger and square-shaped scales, and allow for a closer fitting cell covering. Jadrná et al.^26^ recently reported that scales of the closely related synurophyte genus, *Synura*, have also become smaller and more elongate over geologic time, complementing the observations for *Mallomonas*. Taken together, these findings support the idea that the evolutionary trend for synurophyte organisms has been towards smaller, elliptical scales.

Cyanobacteria, a prokaryotic group of organisms estimated to have evolved by 3.5–3.4 Ga, represent one of the earliest known and smallest life forms on Earth^27^. Since the evolution of these early prokaryotes, Smith et al.^28^ estimated that the maximum body size of subsequent life forms has increased approximately 18-fold, with large jumps occurring with the evolution of eukaryote cells, and another concurrent with the advent of multicellularity. In contrast, shifts in the sizes of siliceous scales and corresponding cells of *Mallomonas* species are small in comparison, within an order of magnitude, and similar to changes observed for prokaryote organisms and other unicellular protists over the Geozoic^28,29^.

Despite the overall lack of historical information on cell size for the majority of unicellular eukaryote lineages, there are data for some organisms that build resistant cell walls or coverings that are taxonomically diagnostic and become incorporated into the fossil record. Diatoms produce a siliceous cell wall known as the frustule, a structure composed of top and bottom pieces called valves that are held together with additional structures called girdle bands. Frustules, or their valve components, can be uncovered from the fossil record and used to provide a direct measure of cell size. Using this technique, Finkel et al.^29^ reported that the size of planktic marine diatoms declined over the Cenozoic, and correlated the shift with abiotic forcing factors, including a rise in sea surface temperature and water column stratification. Foraminifera are heterotrophic marine protists that build shells out of calcium carbonate, the latter of which can also become part of the fossil record. Changes in the size of foraminifera shells over the Cenozoic have also been correlated with shifts in the intensity of water column stratification^30^. At this time, it is not known if the decline in cell size for *Mallomonas* species in the section Planae lineage recorded in the current study was the result of abiotic variables (e.g. energy expenditure or temperature), biotic factors (e.g. predation), or a combination of forcing variables.

The current study has provided a means to link scale size to cell size for *Mallomonas* that, in turn, can be used to trace shifts in cell size over geologic time. As additional scales of *Mallomonas* species are uncovered from the fossil record, the scale-to-cell size model will be a valuable tool for continuing to unravel the evolutionary history of cell size for this important photosynthetic organism. Other groups of unicellular protists, including euglyphids, heliozoids and rotosphaerids, are similar to synurophytes in that they build cell coverings using numerous overlapping siliceous scales or plates that can become fossilize. Perhaps the same technique of relating scale size to cell size could be used to develop models for these protist organisms, and similarly applied to the fossil record.

It is interesting to note that most modern *Mallomonas* species with large body scales are found in warm tropical regions, including *M*. *bronchartiana* Compère, *M*. *pseudobronchartiana* Gusev, Siver & Shin, *M*. *velari* Gusev, Siver & Shin^31^, *M*. *vietnamica* Gusev, Kezlya & Trans^32^, *M*. *gusakovii*^33^ and several varieties of *M*. *matvienkoae*^16^. In addition, the modern tropical taxa *M*. *neoampla* Gusev & Siver and *M*. *vietnamica* share several rare features of their scales and bristles with fossil species recorded from the Giraffe locality, suggesting a possible link between the modern tropical and fossil floras. During the early to middle Eocene, the Earth experienced warm greenhouse conditions and lacked a cryosphere^34^. The Giraffe locality, positioned near the Arctic Circle, had an estimated mean annual temperature 17 °C warmer, and a mean annual precipitation over four times higher, than present conditions^35^. In fact, the assemblage of plants and animals in the Eocene Arctic has been described as analogous to those found today in eastern Asia^36^. Perhaps tropical regions, especially in southeastern Asia, offered refugia for some of the ancient *Mallomonas* lineages.

In summary, multiple extinct fossil species of the diverse and common synurophyte genus, *Mallomonas*, are reported here to have possessed gigantic scales that are significantly larger than those found on modern species. Based on a model relating scale to cell size, cells of fossil *Mallomonas* species were estimated to be, on average, twice as large as modern species. A combination of larger cells with heavy siliceous scales that fit less effectively around the cell may have resulted in slower cells more prone to predation, heavier cells requiring more energy resources to maintain their position in the water column, and ultimately their demise. Additional fossil species, especially representing other localities and time periods, will ultimately strengthen our understanding of the evolution of scale and cell size in synurophyte algae.

## Supplementary Information

Supplementary Information 1.

Supplementary Information 2.

Supplementary Information 3.

## Data Availability

Data supporting this study is given in Supplementary File 1, along with all references used to develop the dataset.

## References

[1] Kristiansen, J. *Golden Algae: A Biology of Chrysophytes* 1–167 (Koeltz Scientific Books, 2005).

[2] Škaloud P, Kristiansen J, Škaloudová M (2013). Developments in the taxonomy of silica-scaled chrysophytes—from morphological and ultrastructural to molecular approaches. Nord. J. Bot..

[3] Siver PA, Wehr JD, Sheath RG, Kociolek JP (2015). The Synurophyceae. Freshwater Algae of North America: Ecology and Classification.

[4] Pusztai M, Škaloud P (2019). Elucidating the evolution and diversity of *Uroglena*-like colonial flagellates (Chrysophyceae): Polyphyletic origin of the morphotype. Eur. J. Phycol..

[5] Kristiansen J, Škaloud P, Archibald JM (2017). Chrysophyta. Handbook of the Protists.

[6] Smol JP, Sandgren CD, Smol JP, Kristiansen J (1995). Application of chrysophytes to problems in paleoecology. Chrysophyte Algae: Ecology, Phylogeny and Development.

[7] Cronberg G (1989). Scaled chrysophytes from the tropics. Beih. Nova Hedwigia.

[8] Cronberg G (1996). Scaled chrysophytes from the Okavango Delta, Botswanam, Africa. Nova Hedwigia Beiheft.

[9] Němcová Y, Kreidlová J, Kosová A, Neustupa J (2012). Lakes and pools of Aquitaine region (France)—a biodiversity hotspot of Synurales in Europe. Nova Hedwigia.

[10] Gusev ES, Doan-Nhu H, Nguyen-Ngoc L, Guseva EE, Phan-Tan L (2019). Silica-scaled chrysophytes from Cam Ranh Peninsula (Khanh Hoa Province, Vietnam). Nova Hedwig. Beih..

[11] Siver PA, Lott AM, Cash E, Moss J, Marsicano LJ (1999). Century changes in Connecticut, U.S.A., lakes as inferred from siliceous algal remains and their relationship to land-use changes. Limnol. Oceanogr..

[12] Arseneau KMA, Driscoll CT, Cummings CN, Pope G, Cumming BF (2016). Adirondack (NY, USA) reference lakes show a pronounced shift in chrysophyte species composition since ca. 1900. J. Paleolimnol..

[13] Siver PA, Wolfe AP (2009). Tropical ochrophyte algae from the Eocene of northern Canada: A biogeographic response to past global warming. Palaios.

[14] Siver PA, Glew JR (1990). The arrangement of scales and bristles on *Mallomonas*: A proposed mechanism for the formation of the cell covering. Can. J. Bot..

[15] Siver PA (1991). The Biology of Mallomonas: Morphology, Taxonomy and Ecology.

[16] Kristiansen J (2002). The genus *Mallomonas* (Synurophyceae)—A taxonomic survey based on the ultrastructure of silica scales and bristles. Opera Bot..

[17] Harris K (1953). A contribution to our knowledge of *Mallomonas*. Bot. J. Linn. Soc..

[18] Siver PA, Wolfe AP (2005). Eocene scaled chrysophytes with pronounced modern affinities. Int. J. Plant Sci..

[19] Siver PA, Jo BY, Kim JI, Shin W, Lott AM, Wolfe AP (2015). Assessing the evolutionary history of the class Synurophyceae (Heterokonta) using molecular, morphometric, and paleobiological approaches. Am. J. Bot..

[20] Siver PA, Lott AM, Wolfe AP (2009). Taxonomic significance of asymmetrical helmet and lance bristles in the genus *Mallomonas* and their discovery in Eocene lake sediments. Eur. J. Phycol..

[21] Siver PA, Wolfe AP (2010). A whole-cell reconstruction of *Mallomonas porifera* Siver and Wolfe from the Eocene: Implications for the evolution of Chrysophyte cell architecture. Nova Hedwig. Beih..

[22] Siver PA (2018). *Mallomonas aperturae* sp. nov (Synurophyceae) reveals that the complex cell architecture observed on modern synurophytes was well established by the middle Eocene. Phycologia.

[23] Škaloud P (2020). Comparing morphological and molecular estimates of species diversity in the freshwater genus *Synura* (Stramenopiles): A potential model for understanding diversity of eukaryotic microorganisms. J. Phycol..

[24] Jo BY, Shin W, Kim HS, Siver PA, Andersen RA (2013). Phylogeny of the genus *Mallomonas* (Synurophyceae) and descriptions of five new species on the basis of morphological evidence. Phycologia.

[25] Reyes, A., *et al*. Prospects, pitfalls, and pratfalls of high latitude paleoenvironmental reconstruction from the post-eruptive sedimentary fill of kimberlite pipes in northern Canada. In *Geological Society of America Annual Conference*, Dec 17 (2020).

[26] Jadrná I, Siver PA, Skaloud P (2020). Morphological evolution of silica scales in the freshwater genus *Synura* (Stramenopiles). J. Phycol..

[27] Schopf JW (2006). The first billion years: When did life emerge?. Elements.

[28] Smith FA (2016). Body size evolution across the geozoic. Annu. Rev. Earth Planet. Sci..

[29] Finkel ZV, Katz ME, Wright JD, Schofield OME, Falkowski PG (2005). Climatically driven macroevolutionary patterns in the size of marine diatoms over the Cenozoic. Proc. Natl. Acad. Sci. U.S.A..

[30] Schmidt DN, Thierstein HR, Bollmann J, Schiebel R (2004). Abiotic forcing of plankton evolution in the cenozoic. Science.

[31] Gusev E, Siver PA, Shin W (2017). *Mallomonas bronchartiana* Compère revisited: Two new species described from Asia. Cryptogam. Algol..

[32] Gusev ES, Kezlya E, Tran H, Kulikovskiy MS (2021). *Mallomonas vietnamica* Gusev, Kezlya & Tran, sp. Nov. (Synurales, Chrysophyceae), a new species, that shares some features with fossil taxa. Cryptogam. Algol..

[33] Gusev ES, Kapustin DA, Martynenko NA, Guseva EE, Kulikovskiy MS (2019). *Mallomonas gusakovii* sp. nov (Chrysophyceae, Synurales), a new species from Phu Quoc Island, Vietnam. Phytotaxa.

[34] Zachos JC, Dickens GR, Zeebe RE (2008). An early Cenozoic perspective on greenhouse warming and carbon-cycle dynamics. Nature.

[35] Wolfe AP, Reyes AV, Royer DL, Greenwood DR, Doria G, Gagen MH, Siver PA, Westgate JA (2017). Middle Eocene CO_2_ and climate reconstructed from the sediment fill of a subarctic kimberlite maar. Geology.

[36] Schubert BA, Jahren AH, Eberle JJ, Sternberg LSL, Eberth DA (2012). A summertime rainy season in the Arctic forests of the Eocene. Geology.

[37] Siver PA (2018). *Mallomonas skogstadii* sp. nov. and *M. bakeri* sp. nov.: Two new fossil species from the middle Eocene representing extinct members of the section Heterospinae?. Cryptog. Algol..

[38] Siver PA, Wolfe AP (2016). *Mallomonas elephantus* sp. nov. (Synurophyceae), an extinct fossil lineage bearing unique scales from the Eocene. Nova Hedwig..

[39] Siver PA, Lott AM (2012). Fossil species of *Mallomonas* from an Eocene Maar lake with recessed dome structures: Early attempts at securing bristles to the cell covering?. Nova Hedwig..

[40] Siver PA, Wolfe AP (2005). Scaled chrysophytes in Middle Eocene lake sediments from Northwestern Canada, including descriptions of six new species. Nova Hedwig. Beih..

[41] Siver PA (2015). *Mallomonas schumachii* sp. nov., a fossil synurophyte bearing large scales described from an Eocene maar lake in Northern Canada. Nova Hedwig..

