# Supplementary material for: The downsizing of gigantic scales and large cells in the genus Mallomonas (Synurales, Chrysophyceae)
Source: Sci Rep. 2022 Mar 22;12:4896. doi: 10.1038/s41598-022-09006-1 (PMC8941141; doi:10.1038/s41598-022-09006-1)
Supplement: Supplementary file 2 — Supplementary Information 2. [file 41598_2022_9006_MOESM2_ESM.docx]

Supplementary Table 1. Scale and cell morphometric data for 144 species of the chrysophyte genus *Mallomonas*. Information for modern and fossil taxa, and references, are given. Cell lengths and widths for fossil species were inferred from a model relating scale size to cell size. See text for details.
