# Supplementary material for: The downsizing of gigantic scales and large cells in the genus Mallomonas (Synurales, Chrysophyceae)
Source: Sci Rep. 2022 Mar 22;12:4896. doi: 10.1038/s41598-022-09006-1 (PMC8941141; doi:10.1038/s41598-022-09006-1)
Supplement: Supplementary file 3 — Supplementary Information 3. [file 41598_2022_9006_MOESM3_ESM.docx]

Supplemental Table 2. Mean length and wide of body scales ± Standard deviations for 21 fossil species.
